# Self-Reported Provision of Community Pharmacy Services and Perceived Barriers in Saudi Arabia: A Cross-Sectional Survey

**DOI:** 10.3390/healthcare14203410

**Published:** 2026-10-13

**Authors:** Emtenan M. Alharbi, Manal M. Alharbi, Amal Qassim Aldarwesh, Moureq Alotaibi, Arwa Omar Bafail, Rawabi Rashed Alsaegh

**Affiliations:** 1 Department of Clinical Pharmacy, College of Pharmacy, King Saud University, P.O. Box 2457, Riyadh 11451, Saudi Arabia; 2 Department of Optometry, College of Applied Medical Sciences, King Saud University, P.O. Box 10219, Riyadh 11433, Saudi Arabia; 3 Department of Pharmacology and Toxicology, College of Pharmacy, King Saud University, P.O. Box 2457, Riyadh 11451, Saudi Arabia; 4 College of Pharmacy, King Saud University, P.O. Box 2457, Riyadh 11451, Saudi Arabia

**Keywords:** community pharmacy, health services, pharmaceutical care, implementation barriers, screening services, Saudi Arabia, cross-sectional study

## Abstract

**Background/Objectives:** Community pharmacists can broaden access to medication-related, preventive, and screening services, but implementation in Saudi Arabia remains uneven. This study described self-reported counselling and extended-service provision, perceived barriers, and statistical relationships between pharmacist- and practice-level characteristics and reported service delivery. **Methods:** A cross-sectional online survey included 600 community pharmacists in Saudi Arabia; an independent pilot included 50 pharmacists. The instrument assessed seven counselling practices, eight extended services, and thirteen barriers. Group comparisons used chi-square or Fisher–Freeman–Halton exact tests with false-discovery-rate adjustment, and threshold-specific logistic regression models evaluated service provision. **Results:** Agreement with counselling practices ranged from 78.7% to 91.3%. Frequent provision was low for vaccination (6.0%), cholesterol screening (8.2%), glucose screening (10.5%), and blood pressure assessment (11.7%). Leading barriers were insufficient public awareness (66.3%), limited patient time (64.5%), lack of medical-record access (58.3%), inadequate privacy (56.5%), and weak primary-care integration (54.7%). Educational qualification did not show an independent adjusted relationship with extended-service provision. Selected service outcomes differed across practice-setting categories, and exploratory analyses identified an inverse statistical relationship between perceived barriers and extended-service provision. **Conclusions:** Routine counselling was commonly reported, whereas most extended services were infrequent. Expanding community pharmacy services requires attention to workflow, staffing, privacy, patient information, public awareness, referral pathways, and health-system integration.

## 1. Introduction

Community pharmacies are among the most accessible points of healthcare and increasingly complement the medication supply with patient counselling, medication-use support, health promotion, screening, vaccination, and chronic disease services [1,2,3,4,5,6]. Their accessibility creates opportunities to support self-care, earlier intervention, and continuity of care; however, the extent to which these services are routinely implemented varies considerably across health systems.

International evidence indicates that implementation depends not only on pharmacists’ clinical competence but also on workflow, staffing, remuneration, privacy, access to patient information, digital infrastructure, interprofessional communication, and public awareness [7,8,9,10,11]. These factors become particularly important as community pharmacy practice moves beyond traditional dispensing towards structured, patient-centred service delivery.

Recent evidence from Saudi Arabia has further clarified both the opportunity and the implementation gap. Public-perspective research has identified a limited awareness of the full range of community pharmacy clinical services and the continued reliance on other healthcare professionals [12]. A 2025 systematic review of 89 Saudi studies published from 2010 to 2020 reported deficiencies in patient counselling, the limited knowledge and provision of clinical services, the under-representation of several key stakeholder perspectives, and a need for stronger integration across the commercial, administrative, and technical sectors [13]. This review therefore provides an important pre-2021 baseline but does not characterise the contemporary pharmacist-reported implementation of multiple service domains in the post-2020 period. More recent pharmacist-focused studies have examined specific domains, including minor-ailment services, medication counselling, asthma management, and drug–drug interaction management; these studies repeatedly identified limited patient information, inadequate private counselling space, insufficient training or reimbursement, time pressure, workload, and weak interprofessional communication as barriers [14,15,16,17,18].

Saudi pharmacy education includes both the Bachelor of Pharmacy (B.Pharm) and Doctor of Pharmacy (PharmD) pathways. Contemporary Saudi literature describes the B.Pharm as a five-year professional programme with greater emphasis on pharmaceutical sciences, whereas the PharmD is typically a six-year entry-level programme with greater emphasis on clinical practice and internship training [19,20]. Both B.Pharm and PharmD graduates may qualify for pharmacist practice in Saudi Arabia after meeting the Saudi Commission for Health Specialties licensure requirements; however, the degree title alone should not be interpreted as establishing clinical-pharmacist status, which requires additional postgraduate education or training [19]. In the present study, the degree title was therefore treated as a broad educational characteristic; the survey did not verify the awarding institution, individual curriculum, postgraduate training, or licensure category for each respondent.

However, recent Saudi studies have generally been public-facing, regional, qualitative, or focused on a single clinical service. Consequently, contemporary quantitative evidence remains limited on the simultaneous breadth of routine counselling, screening, preventive and other extended services, the barriers perceived across these domains, and the pharmacist- and practice-level characteristics associated with their provision within one Saudi community-pharmacy sample. Accordingly, this study aimed to describe self-reported community pharmacy service provision in Saudi Arabia, identify perceived implementation barriers, and examine statistical relationships with pharmacist- and practice-level characteristics. The study was descriptive and exploratory; no directional a priori hypothesis was specified, and the adjusted analyses were intended to characterise non-causal statistical associations rather than test a causal model.

## 2. Materials and Methods

### 2.1. Study Design and Participants

This cross-sectional study used an English-language, self-administered online questionnaire hosted on Google Forms (Google LLC, Mountain View, CA, USA) and targeted pharmacists practising in community pharmacies in Saudi Arabia. Data collection began on 24 October 2023, after IRB approval on 15 October 2023, and continued until 15 January 2024. Recruitment used a multichannel non-probability convenience approach. Pharmacy administrators and professional contacts at three major community-pharmacy chains operating in Saudi Arabia—Nahdi Medical Company, Al-Dawaa Pharmacies, and United Pharmacy—were approached by email and telephone and asked to distribute the survey to eligible community pharmacists. The survey was also distributed directly through field visits and through colleagues, students, and professional contacts. Eligibility was managed during recruitment: managers/administrators and other distributors were instructed to provide the survey only to eligible community pharmacists, while direct recruiters approached pharmacists working at community-pharmacy branches. No pharmacist name, licence number, or other direct personal identifier was collected for recruitment control. Recruitment was controlled at the pharmacy-branch level. Each accepted response carried a pharmacy branch number/code that was recorded separately for recruitment control and was not a participant-facing questionnaire item. For direct distribution routes, including field visits and distribution through colleagues, students, and professional contacts, the recruiter knew the branch and checked or recorded its branch number/code to ensure that only one response was obtained from that branch. When the survey was distributed through company managers/administrators or another contact to a different branch, the distributor was instructed to obtain only one response from that branch and to notify the research team if more than one response was inadvertently completed. No duplicate branch code was reported to or identified by the research team during data collection, so no duplicate-resolution procedure was required. The branch code identified the pharmacy branch only and contained no pharmacist name or licence number. It was used solely for recruitment control and was removed before preparation of the de-identified analytical dataset; it was not available to the statistician. Under this procedure, the analytical sample represented no more than one response per recorded pharmacy branch. The form was also configured to permit one response per Google account. Because the survey was circulated through several channels and the total number of pharmacists who received the invitation was not documented, a conventional response rate could not be calculated; the achieved sample size should therefore not be interpreted as a response rate. The non-probability recruitment strategy also did not define a national sampling frame.

### 2.2. Survey Instrument

The questionnaire contained three sections. Section S1 (File S1) collected demographic and professional characteristics: gender, age group, nationality, educational qualification, years of community pharmacy experience, average daily customer volume, pharmacy location, and pharmacy type. Sections S2A and S2B (File S1) assessed seven medication-counselling practices using a five-point agreement scale and eight extended health services using a five-point frequency scale ranging from never to always. Section S3 (File S1) assessed thirteen patient-, pharmacist-, organisational-, and health-system-related barriers using a five-point agreement scale. The instrument was developed from relevant literature and underwent expert review by knowledgeable colleagues and a statistician to assess clarity, comprehensibility, and relevance. The questionnaire content and response options are presented in Supplementary File S1, and the consent statement and participant-facing survey layout are provided in Supplementary File S4.

### 2.3. Pilot Testing and Internal Consistency

Before the main survey, the questionnaire was pilot-tested among 50 community pharmacists to assess clarity, usability, and internal consistency. The pilot participants were not included in the final analytical sample of 600 pharmacists. Pilot feedback resulted only in minor formatting modifications, with no substantive changes to the items, domains, response options, or intended meanings. Cronbach’s alpha was 0.917 for the seven counselling-practice items, 0.860 for the eight extended-service items, and 0.865 for the thirteen perceived-barrier items, indicating good to excellent internal consistency. Internal consistency was reassessed in the final analytical sample and remained high.

### 2.4. Eligibility and Sample Size

Eligible participants were qualified pharmacists currently working in a community pharmacy in Saudi Arabia, willing to participate, and able to understand the English-language questionnaire. Eligibility was managed during recruitment as described in Section 2.1. Managers/administrators and other distributors were instructed to provide the survey only to eligible community pharmacists, and direct recruiters approached pharmacists working at community-pharmacy branches. Each accepted response carried a pharmacy branch number/code for branch-level recruitment control; no pharmacist name, licence number, or other direct personal identifier was collected. Pharmacists not working in community pharmacy, those unable to understand the questionnaire, and those who did not consent were excluded. Using a conservative expected proportion of 50%, a 95% confidence level, and a 5% margin of error, the minimum required sample was 385 completed questionnaires. The achieved analytic sample was 600 pharmacists.

### 2.5. Ethical Considerations

The study was approved by the King Saud University Institutional Review Board (Project No. E-23-8203; Ref. No. 23/0769/IRB) on 15 October 2023. All participants received study information electronically and provided informed consent before accessing the questionnaire. Participation was voluntary, and survey responses were collected without names, licence numbers, or other direct personal identifiers. A pharmacy branch number/code accompanied each accepted response solely for recruitment control. The code identified the pharmacy branch rather than the individual pharmacist and was removed before preparation of the de-identified analytical dataset; it was not available to the statistician.

### 2.6. Statistical Analysis

The original text responses exported from Google Forms were independently recoded according to the questionnaire response categories and compared with the archived coded dataset. Categorical variables were summarised as frequencies and percentages. Internal consistency was evaluated separately for each questionnaire domain using Cronbach’s alpha, standardised alpha, corrected item–total correlations, alpha if item deleted, and 95% bootstrap confidence intervals based on 2000 resamples. Educational-group comparisons used Pearson’s chi-square test when expected-cell assumptions were satisfied and the Fisher–Freeman–Halton exact test with 100,000 Monte Carlo samples when sparse cells were present. Bias-corrected Cramér’s V was calculated as an effect-size measure. Benjamini–Hochberg false-discovery-rate adjustment was applied separately within the counselling, extended-service, and barrier domains; adjusted values are reported as q-values. For adjusted service-specific analyses, two threshold-specific binary logistic regression models were fitted for each extended service: (1) any provision (occasionally, sometimes, mostly, or always) versus never, and (2) frequent provision (mostly or always) versus less frequent provision. This approach avoided imposing a common proportional-odds effect across thresholds. Models included age group, gender, nationality, educational qualification, average daily customer volume, location inside or outside Riyadh, and independent or chain pharmacy type. Location was modelled using the primary survey item (inside Riyadh versus outside Riyadh), reflecting the primary geographic comparison of the national capital with locations elsewhere in Saudi Arabia. The questionnaire also collected follow-up North/South/East/West location categories. In a post hoc geographic sensitivity analysis of the any-provision models, the binary location term was replaced by four outside-Riyadh directional categories (West, North, East, and South), each compared with Riyadh. The same covariates as in the primary adjusted models were retained; respondents without usable outside-Riyadh directional data were excluded from this sensitivity analysis, and Benjamini–Hochberg adjustment was applied across the 32 directional contrasts. Models were specified as main-effects models; interaction terms were not prespecified or tested because the study was descriptive/exploratory and several frequent-service outcomes contained sparse cells. Age was entered as categorical indicators to avoid imposing a linear trend, and years of experience was not entered simultaneously because of its overlap with age. Variance-inflation factors were below 5. Three chain-pharmacy coefficients at the frequent-provision threshold (vaccination, cholesterol screening, and glucose screening) were affected by quasi-complete separation because no frequent-service events were observed among independent-pharmacy respondents for these outcomes. Firth penalised logistic regression was therefore performed as a post hoc sensitivity analysis using the same adjusted covariate set. Although Firth estimation yielded finite coefficients, the resulting confidence intervals remained extremely wide, indicating substantial residual imprecision due to sparse data. The coefficients were therefore retained as not estimable in the primary maximum-likelihood framework, and the Firth estimates were treated as sensitivity analyses rather than replacements for the common primary modelling approach. The Firth sensitivity estimates are reported in Supplementary File S3, Table S5A. Benjamini–Hochberg adjustment was applied separately across the 88 slope tests within each threshold family. Exploratory domain-level analyses used Spearman’s rank correlation and multivariable linear regression with heteroskedasticity-consistent HC3 standard errors. Two-sided q-values < 0.05 were used for multiplicity-adjusted inference; exploratory analyses are reported with two-sided *p*-values. Analyses were finalised using Python 3.13 (Python Software Foundation, Beaverton, OR, USA), with SciPy 1.17.0 and statsmodels 0.14.6. The authors specified all analytical decisions, reviewed the analytical outputs, cross-checked the results against the archived analyses, and take full responsibility for the reported findings. Reporting followed the STROBE recommendations for cross-sectional studies [21]; the completed checklist is provided as Supplementary File S2. Detailed statistical outputs are provided in Supplementary File S3.

## 3. Results

### 3.1. Participant Characteristics

The analytical sample comprised 600 community pharmacists: 87 (14.5%) held a PharmD and 513 (85.5%) held a B.Pharm. PharmD respondents were younger, more frequently female and Saudi, and had fewer years of community pharmacy experience than B.Pharm respondents (all *p* < 0.001). Customer volume also differed between groups (*p* = 0.002). Pharmacy location (*p* = 0.833) and pharmacy type (*p* = 0.494) did not differ by educational qualification (Table 1). All 600 respondents had complete data for the 28 counselling, extended-service, and barrier items.

In the final sample, Cronbach’s alpha was 0.930 (95% bootstrap CI 0.915–0.942) for counselling practices, 0.886 (95% CI 0.865–0.903) for extended services, and 0.932 (95% CI 0.920–0.942) for perceived barriers. Corrected item–total correlations ranged from 0.486 to 0.834, and deleting any item did not materially improve its domain alpha (Supplementary File S3, Table S2).

### 3.2. Self-Reported Counselling Practices

Agreement with the seven counselling-practice statements was high overall, ranging from 78.7% for medication-error identification and reporting to 91.3% for counselling with every dispensing. After false-discovery-rate adjustment, response distributions differed by educational qualification for counselling with every dispensing (q = 0.019; Cramér’s V = 0.147) and detection of drug–drug interactions or adverse drug reactions (q = 0.019; Cramér’s V = 0.138). Agreement or strong agreement was lower among PharmD than B.Pharm respondents for both items (83.9% versus 92.6%, and 69.0% versus 83.8%, respectively). No other counselling item differed after adjustment (Table 2; Supplementary File S3, Table S3).

### 3.3. Extended Health-Service Provision

Extended health services were reported less frequently than routine counselling. Frequent provision (mostly or always) was reported for vaccination by 6.0% of respondents, blood cholesterol screening by 8.2%, blood glucose screening by 10.5%, blood pressure assessment by 11.7%, body mass index assessment by 12.5%, emergency contraception service by 18.8%, smoking-cessation counselling by 19.3%, and herbal-medicine advice by 34.8%. None of the educational-group comparisons remained significant after false-discovery-rate adjustment (Table 3).

In the adjusted threshold-specific logistic regression models, educational qualification did not show an FDR-significant relationship with any of the eight services at either threshold (all q ≥ 0.172). Higher adjusted odds of any provision, rather than never, were observed outside Riyadh for vaccination (aOR 2.40, 95% CI 1.65–3.48), body mass index assessment (aOR 3.71, 95% CI 2.52–5.46), cholesterol screening (aOR 2.78, 95% CI 1.92–4.03), glucose screening (aOR 3.71, 95% CI 2.56–5.38), blood pressure assessment (aOR 3.43, 95% CI 2.37–4.96), and smoking-cessation counselling (aOR 2.02, 95% CI 1.33–3.07; all q < 0.016). Higher adjusted odds were also observed in chain pharmacies for any cholesterol screening (aOR 3.06, 95% CI 1.55–6.06; q = 0.017) and any blood pressure assessment (aOR 2.61, 95% CI 1.38–4.93; q = 0.030), and among pharmacies serving more than 100 customers daily for any blood pressure assessment (aOR 2.42, 95% CI 1.38–4.23; q = 0.022). At the frequent-provision threshold, chain-pharmacy practice showed higher adjusted odds of frequent herbal-medicine advice (aOR 8.26, 95% CI 3.47–19.66; q < 0.001). Two additional estimates met the FDR threshold but were imprecise: chain-pharmacy practice with frequent smoking-cessation counselling (aOR 10.65, 95% CI 2.53–44.88; q = 0.046) and female gender with frequent emergency-contraception service (aOR 2.98, 95% CI 1.51–5.86; q = 0.046). The wide confidence intervals indicate substantial uncertainty, so these estimates are not emphasised in the interpretation or conclusions. Full models are presented in Supplementary File S3, Table S1. These cross-sectional estimates describe statistical relationships and should not be interpreted as causal effects.

Post hoc Firth sensitivity analyses for the three separated chain-pharmacy coefficients produced finite but highly imprecise estimates: vaccination (aOR 9.17, 95% penalised profile-likelihood CI 1.23–1170.36), cholesterol screening (aOR 12.79, 95% CI 1.75–1629.77), and glucose screening (aOR 16.27, 95% CI 2.25–2067.88). The extremely wide confidence intervals indicate substantial residual imprecision, so these estimates were treated as sensitivity analyses only (Supplementary File S3, Table S5A).

Usable outside-Riyadh directional data were available for 237 of 249 respondents; together with all 351 Riyadh respondents, the post hoc geographic sensitivity analysis included 588 participants. Relative to Riyadh, all four outside-Riyadh directional groups showed higher adjusted odds of any BMI assessment (aOR range 3.52–5.76), glucose screening (aOR range 2.90–4.68), and blood-pressure assessment (aOR range 2.74–4.76), with all corresponding contrasts remaining significant after false-discovery-rate adjustment. Vaccination and cholesterol-screening estimates were also greater than 1.0 in all four directional groups, with three of four contrasts for each outcome remaining significant after adjustment. Patterns for smoking-cessation counselling, emergency contraception, and herbal-medicine advice were less consistent (Supplementary File S3, Table S5B).

### 3.4. Perceived Barriers to Service Provision

The most frequently perceived barriers were an insufficient public awareness of available services (66.3% agreement or strong agreement), limited patient time (64.5%), lack of access to patient medical records (58.3%), inadequate privacy space (56.5%), insufficient integration with primary healthcare (54.7%), limited patient demand (52.3%), insufficient pharmacist time (51.5%), and shortages of pharmacy assistants or technicians (51.2%).

After false-discovery-rate adjustment, educational-group response distributions differed for insufficient coordination with other healthcare professionals (q = 0.045; Cramér’s V = 0.124), lack of access to patient medical records (q < 0.001; Cramér’s V = 0.195), and insufficient training (q = 0.037; Cramér’s V = 0.133). Agreement or strong agreement with these barriers was reported by 52.9%, 69.0%, and 60.9% of PharmD respondents, respectively, compared with 49.3%, 56.5%, and 44.4% of B.Pharm respondents. Other barrier comparisons did not remain significant after adjustment (Table 4; Supplementary File S3, Table S3).

### 3.5. Exploratory Domain-Level Analyses

The mean extended-service score was inversely correlated with the mean perceived-barrier score (Spearman’s ρ = −0.312, *p* < 0.001), whereas counselling was not significantly correlated with either extended-service provision or perceived barriers. In an exploratory robust linear model adjusted for the demographic and practice characteristics used in the service-specific models and for the counselling score, each one-point increase in the mean barrier score corresponded to a 0.282-point lower extended-service score (95% CI −0.394 to −0.170; *p* < 0.001; model R^2^ = 0.200). Full exploratory domain-level results are presented in Supplementary File S3, Table S4.

## 4. Discussion

### 4.1. Principal Findings

This survey of 600 community pharmacists provides a broad assessment of routine counselling practices, extended health-service provision, and perceived implementation barriers in Saudi Arabia. Routine medication counselling was commonly reported, whereas vaccination and most screening services were provided infrequently. The high self-reported counselling levels require cautious interpretation because they contrast with objective simulated-patient evidence from Saudi Arabia: in 134 unannounced encounters involving potential drug–drug interactions, only 14.9% of pharmacists recognised the interaction and 13.4% provided clear risk counselling [22]. The present survey therefore reflects reported practice rather than directly observed performance. The leading perceived barriers were insufficient public awareness, limited patient and pharmacist time, lack of access to medical records, inadequate privacy, and weak integration with primary healthcare. Educational qualification did not show a statistically significant adjusted relationship with extended-service provision. Differences in selected outcomes were observed across location, pharmacy organisation, and customer-volume categories, and exploratory analysis showed an inverse statistical relationship between overall barrier scores and extended-service provision. The overall pattern suggests an implementation gap, but the cross-sectional design does not establish causal effects.

### 4.2. Comparison with Saudi and Regional Evidence

Agreement with routine counselling-practice statements was high, ranging from 78.7% for medication-error identification and reporting to 91.3% for counselling with every dispensing. These findings appear more favourable than the 2025 systematic review of 89 Saudi studies from 2010–2020, which identified deficient patient counselling, limited knowledge and delivery of clinical services, and gaps in stakeholder integration [13]. This difference should not be interpreted as proof of improvement over time because the underlying studies used different populations, measures, and data-collection methods. In particular, self-reported agreement with a practice statement may overestimate what is consistently delivered or understood by patients. Nevertheless, the present findings indicate that medication counselling is recognised as part of routine community pharmacy practice, even when more structured clinical services remain limited.

The distinction between routine counselling and more complex safety communication is consistent with a 2026 study of community pharmacists in Jazan. That study found the near-universal provision of core medicine-use instructions but lower risk communication, and identified the presence of pharmacy support staff—not the PharmD qualification—as a principal determinant of risk counselling [16]. Similarly, the present adjusted analyses did not show an independent advantage of PharmD education for extended service provision. The comparison supports a practical interpretation: advanced education may expand competence, but pharmacists need sufficient time, staffing, information, and workflow support to translate competence into routine service delivery.

The high self-reported frequency of detecting drug–drug interactions and adverse drug reactions in the present survey should also be interpreted separately from objectively assessed knowledge. A 2026 Jazan study that tested the knowledge of clinically relevant drug pairs found moderate overall performance and identified time pressure, limited patient understanding, and poor collaboration with prescribers as barriers [18]. Thus, the reported involvement in interaction screening does not necessarily establish the accuracy or completeness of that activity. Future evaluations should combine self-report with case-based knowledge assessment, dispensing records, or simulated-patient methods.

Extended services were much less established than routine counselling. Frequent provision was reported by only 6.0% of respondents for vaccination, 8.2% for cholesterol screening, 10.5% for glucose screening, and 11.7% for blood pressure assessment. This pattern is consistent with the 2025 Gulf Cooperation Council scoping review, which concluded that dispensing and counselling were the most established community pharmacy functions, whereas vaccination, screening, chronic disease monitoring, and other extended services were often locally practised, piloted, or insufficiently evaluated [23]. A 2026 study of asthma services in the Aseer region similarly found that patient and medication education were commonly reported, while written action plans, formal symptom-control assessment, and scheduled follow-up were less frequent [17]. Together, these studies indicate that the principal gap is not the recognition of pharmacists’ clinical role, but the conversion of individual activities into structured, repeatable services. The objective simulated-patient evidence discussed above further reinforces the need to interpret self-reported counselling activity cautiously and supports the use of practice-based evaluation methods.

### 4.3. Perceived Barriers and Practice-Setting Associations

The barrier findings were highly consistent with recent Saudi research across different service areas. In a 2025 national study of readiness for minor-ailment services, the leading barriers included a lack of reimbursement, insufficient staffing, lack of training, weak public awareness, and limited collaboration with physicians [14]. In a 2026 Saudi asthma study, pharmacists reported time pressure, lack of private counselling areas, low patient awareness, and limited access to patient information as important constraints [17]. The inverse statistical relationship between the overall barrier score and extended-service provision in the present study is consistent with the interpretation that these barriers are operational rather than merely attitudinal. The convergence of findings across general, minor-ailment, and disease-specific services suggests that the barriers are not confined to a particular clinical programme.

Qualitative evidence also supports this interpretation. Stakeholders in Al Baha described community pharmacy practice as predominantly dispensing-oriented and identified public awareness, privacy and confidentiality, and weak communication between pharmacists and physicians as central barriers to integrating extended services into the healthcare system [15]. At an international level, a 2025 scoping review of implemented pharmacist-led services in Organisation for Economic Co-operation and Development countries identified time and workload pressure, limited consumer awareness, resistance from other healthcare professionals, and remuneration concerns as recurrent implementation barriers. Clear role definitions, structured service-delivery models, collaboration, motivated staff, and financial incentives were recurrent facilitators [11]. The consistency across contexts strengthens the argument that community pharmacy expansion requires coordinated changes at the patient, pharmacy, professional, and health-system levels rather than isolated educational interventions. A 2026 Saudi mixed-methods SWOT analysis similarly identified infrastructure limitations, workforce challenges, regulatory fragmentation, and limited clinical integration as constraints on expanding community pharmacy services [24]. A 2026 Aseer study of community pharmacists’ diabetes management during Ramadan similarly identified time constraints (85.7%), lack of updated training (84.7%), and inadequate counselling privacy (79.8%) as the leading reported barriers [25]. Similarly, a 2026 five-region Saudi study of 309 community pharmacy staff focused on cancer counselling identified lack of formal training (74.1%), time constraints (69.9%), and insufficient access to up-to-date guidelines (63.7%) as prominent barriers [26].

Higher adjusted odds of any provision of six extended services were observed among respondents practising outside Riyadh. Higher adjusted odds of selected outcomes were also observed in chain pharmacies and in pharmacies with a higher customer volume. The threshold-specific results indicate that characteristics accompanying service initiation may differ from those accompanying frequent provision. These statistical relationships may reflect differences in equipment, staffing, organisational protocols, local demand, or opportunities to incorporate services into routine workflow; they should not be interpreted as causal effects of geography, pharmacy ownership, or customer volume. The broad “outside Riyadh” category encompasses heterogeneous geographic settings. However, the post hoc directional sensitivity analysis showed that the higher any-provision estimates for the BMI assessment, glucose screening, and blood-pressure assessment were observed across all four outside-Riyadh directional groups, while the vaccination and cholesterol-screening patterns were also distributed across several groups rather than being driven by a single outside area. These directional categories did not identify specific cities or locality types and were affected by some skip-pattern inconsistency; they should therefore be interpreted as a sensitivity check rather than as a direct comparison of individual Saudi regions.

Recent evidence shows that knowledge and service delivery may follow different organisational patterns. In the Aseer asthma study, pharmacists in independent pharmacies had higher knowledge, whereas chain pharmacists reported higher practice and barrier scores [17]. The Jazan counselling study found that support staff were important for risk communication [16]. These observations are compatible with the present findings: individual knowledge may be necessary, but service provision can be more strongly shaped by the operational bandwidth, equipment, staffing, and standardised organisational processes. The absence of an FDR-significant adjusted relationship between educational qualification and either service threshold should not be interpreted as evidence that education is unimportant. Rather, qualification alone appears insufficient to overcome practice-level constraints. The remaining educational-group differences in selected counselling and barrier items were small in magnitude and should be interpreted cautiously because PharmD and B.Pharm respondents differed substantially in age, nationality, gender, and professional experience.

### 4.4. Implications for Community Pharmacy and Healthcare Delivery

The findings support an implementation-focused strategy for expanding community pharmacy services in Saudi Arabia. Training remains important, particularly for service-specific assessment, referral, documentation, and safety; however, training without changes to the practice environment is unlikely to produce sustained delivery. A coordinated service model should combine public communication about pharmacists’ roles, protected consultation space, adequate pharmacist and technician staffing, standardised clinical protocols, documentation and quality-assurance procedures, and formal referral pathways with physicians and primary healthcare services. Appropriate, role-based access to relevant patient information would be particularly important for medication review, interaction assessment, chronic disease support, and follow-up.

Reimbursement was not measured in the present questionnaire and is therefore considered only as contextual evidence. Prior Saudi and international studies have identified reimbursement or remuneration as an implementation barrier [11,14]; this issue warrants direct measurement in future implementation research rather than inference from the present data. Initial implementation could focus on defined, protocol-driven services with a plausible public-health benefit—such as blood pressure and diabetes risk assessment, vaccination, smoking-cessation support, and minor-ailment triage—and should include both chain and independent pharmacies in Riyadh and other regions.

The evaluation should extend beyond pharmacist attitudes and self-reported frequency. Pragmatic pilots should measure the service uptake, referral appropriateness, medication safety, patient-reported experience, clinical outcomes, equity of access, and cost. Standardised service definitions would also improve the comparability across studies, because the same label may currently represent anything from brief advice to a documented consultation with an assessment and follow-up. This shift from measuring willingness to evaluating the implementation and outcomes would provide evidence that is directly relevant to health-service planning and the integration of community pharmacies into primary care. Recent simulated-patient evidence from three Saudi regions further illustrates the importance of measuring the observed service quality: across 251 insomnia-related encounters, the median consultation lasted 1.5 min, safety information was provided infrequently, and privacy and referral practices were limited [27].

### 4.5. Strengths, Limitations, and Future Research

This study has several strengths. The sample of 600 pharmacists exceeded the minimum sample-size requirement and permitted the analysis of a broad range of counselling practices, extended services, and perceived barriers within one survey. An independent pilot sample supported the internal consistency of all three domains, and the final data were recoded directly from the original text responses and cross-checked against the archived coded dataset. The analyses used exact testing when sparse cells were present, false-discovery-rate adjustment, effect-size estimates, and threshold-specific adjusted models that did not impose the proportional-odds assumption. The questionnaire content, participant-facing survey format, STROBE checklist, and detailed statistical tables are supplied as Supplementary Materials, improving transparency and reproducibility.

The findings should nevertheless be interpreted within several limitations. The cross-sectional design precludes causal inference, and self-reported responses may be affected by the recall and social-desirability bias. Electronic non-probability convenience recruitment, the absence of a documented invitation denominator, and the lack of a defined national sampling frame may have introduced selection bias and limit representativeness. Eligibility was managed through the controlled recruitment channels described above. A non-personal pharmacy branch number/code was required for recruitment control and was removed before analysis; because no pharmacist names, licence numbers, or other direct personal identifiers were collected, the individual respondent identity cannot be retrospectively reverified from the analytical dataset. The questionnaire was administered only in English, which may have preferentially selected pharmacists with a greater English proficiency and may have affected participation or response interpretation among both Saudi and expatriate pharmacists. The study-specific instrument showed strong internal consistency, but this does not establish unidimensionality, construct validity, or criterion validity. Service delivery was not verified using pharmacy records, observation, simulated patients, or patient reports, and the study did not assess service quality, clinical outcomes, or economic impact. The primary inside/outside-Riyadh measure combined heterogeneous settings outside the capital. Although a post hoc North/South/East/West sensitivity analysis supported the robustness of several key any-provision findings, these broad directional categories did not identify specific cities or locality types, and some conditional sublocation fields were completed inconsistently; therefore, detailed city- or locality-level geographic inference was not possible. Thresholding service-frequency responses improved interpretability and avoided a proportional-odds assumption but reduced response detail. Sparse frequent-service cells also prevented the stable maximum-likelihood estimation of three chain-pharmacy coefficients, which were reported as not estimable in the primary models and excluded from primary interpretation; post hoc Firth sensitivity estimates were finite but remained highly imprecise because of the extremely wide confidence intervals (Supplementary File S3, Table S5A). The interaction effects were not evaluated, and the residual confounding, unmeasured effect modification, or model misspecification cannot be excluded. The exploratory domain-score analysis should therefore be interpreted as supportive rather than confirmatory.

The questionnaire captured several important barriers but did not directly measure reimbursement, regulatory clarity, accreditation, digital interoperability, or organisational readiness; these factors have emerged prominently in recent Saudi and international studies [11,14,15]. Future research should use regionally stratified probability sampling and psychometrically validated instruments, with objective measures of service delivery obtained from records, direct observation, or simulated-patient methods. Mixed-methods implementation studies should examine how staffing, workflow redesign, private consultation areas, access to patient information, referral pathways, accreditation, and reimbursement influence both the initiation and routine frequency of services. Longitudinal evaluations should incorporate patient and physician perspectives, equity of access, clinical outcomes, and economic consequences to determine whether expanded community pharmacy services produce sustained value for the wider healthcare system.

## 5. Conclusions

This cross-sectional survey found that community pharmacists commonly reported routine medication-counselling practices, whereas vaccination and most screening services were provided infrequently. Insufficient public awareness, limited time, lack of access to medical records, inadequate privacy, and weak integration with primary healthcare were prominent perceived barriers. Educational qualification did not independently explain whether extended services were provided or provided frequently. Differences in selected service outcomes were observed across practice-setting and organisational categories, and exploratory analyses showed an inverse statistical relationship between overall perceived barriers and extended-service provision. Because recruitment was non-probability and the outcomes were self-reported, selection and reporting biases may limit generalisability; the estimates should not be interpreted as nationally representative or causal.

These findings support an implementation-focused approach to community pharmacy expansion. Priorities include public communication about pharmacists’ roles, protected consultation space, adequate staffing, appropriate access to patient information, structured referral pathways, interprofessional collaboration, and sustainable commissioning arrangements. Interventions should distinguish between enabling pharmacies to initiate a service and supporting its regular delivery, and they should be evaluated through pragmatic implementation studies before conclusions are drawn about effectiveness, equity, or cost-effectiveness.

## Figures and Tables

**Table 1:** Participant characteristics according to educational qualification.

Characteristic	PharmD (n = 87)	B.Pharm (n = 513)	*p*-Value
**Age group**			<0.001
20–25	28 (32.2%)	29 (5.7%)	
26–30	36 (41.4%)	150 (29.2%)	
31–35	13 (14.9%)	180 (35.1%)	
36–40	6 (6.9%)	90 (17.5%)	
>40	4 (4.6%)	64 (12.5%)	
**Gender**			<0.001
Female	42 (48.3%)	53 (10.3%)	
Male	45 (51.7%)	460 (89.7%)	
**Nationality**			<0.001
Saudi	70 (80.5%)	83 (16.2%)	
Non-Saudi	17 (19.5%)	430 (83.8%)	
**Years of community pharmacy experience**			<0.001
1–5	60 (69.0%)	105 (20.5%)	
6–10	15 (17.2%)	191 (37.2%)	
11–15	7 (8.1%)	139 (27.1%)	
>16	5 (5.8%)	78 (15.2%)	
**Average daily customer count**			0.002
1–50	33 (37.9%)	118 (23.0%)	
51–100	38 (43.7%)	216 (42.1%)	
>100	16 (18.4%)	179 (34.9%)	
**Location of current work**			0.833
Inside Riyadh	50 (57.5%)	301 (58.7%)	
Outside Riyadh	37 (42.5%)	212 (41.3%)	
**Type of community pharmacy**			0.494
Independent	15 (17.2%)	74 (14.4%)	
Chain	72 (82.8%)	439 (85.6%)	

**Table 2:** Self-reported counselling practices according to educational qualification.

Counselling-Practice Statement	PharmD (n = 87)	B.Pharm (n = 513)	*p*-Value	q-Value
**I provide patient counselling with every dispensing.**			0.005	0.019
Strongly disagree	0 (0.0%)	11 (2.1%)		
Disagree	3 (3.4%)	3 (0.6%)		
Neither agree nor disagree	11 (12.6%)	24 (4.7%)		
Agree	33 (37.9%)	230 (44.8%)		
Strongly agree	40 (46.0%)	245 (47.8%)		
**I provide patients with instructions on drug administration, dosage, side effects, and precautions.**			0.061	0.119
Strongly disagree	2 (2.3%)	8 (1.6%)		
Disagree	2 (2.3%)	11 (2.1%)		
Neither agree nor disagree	15 (17.2%)	42 (8.2%)		
Agree	27 (31.0%)	217 (42.3%)		
Strongly agree	41 (47.1%)	235 (45.8%)		
**I detect drug–drug interactions and adverse drug reactions during counselling.**			0.004	0.019
Strongly disagree	1 (1.1%)	7 (1.4%)		
Disagree	9 (10.3%)	18 (3.5%)		
Neither agree nor disagree	17 (19.5%)	58 (11.3%)		
Agree	27 (31.0%)	234 (45.6%)		
Strongly agree	33 (37.9%)	196 (38.2%)		
**I assist in identifying and reporting medication errors to the appropriate authority.**			0.215	0.251
Strongly disagree	3 (3.4%)	9 (1.8%)		
Disagree	5 (5.7%)	24 (4.7%)		
Neither agree nor disagree	18 (20.7%)	69 (13.5%)		
Agree	28 (32.2%)	217 (42.3%)		
Strongly agree	33 (37.9%)	194 (37.8%)		
**I provide advice on drug therapy for chronic non-communicable diseases.**			0.068	0.119
Strongly disagree	1 (1.1%)	7 (1.4%)		
Disagree	6 (6.9%)	19 (3.7%)		
Neither agree nor disagree	16 (18.4%)	49 (9.6%)		
Agree	30 (34.5%)	222 (43.3%)		
Strongly agree	34 (39.1%)	216 (42.1%)		
**I provide counselling on non-prescription options for the common cold or influenza.**			0.318	0.318
Strongly disagree	1 (1.1%)	6 (1.2%)		
Disagree	5 (5.7%)	20 (3.9%)		
Neither agree nor disagree	12 (13.8%)	45 (8.8%)		
Agree	26 (29.9%)	205 (40.0%)		
Strongly agree	43 (49.4%)	237 (46.2%)		
**I provide patients with medical information about diseases and medications.**			0.090	0.126
Strongly disagree	3 (3.4%)	10 (1.9%)		
Disagree	6 (6.9%)	18 (3.5%)		
Neither agree nor disagree	13 (14.9%)	47 (9.2%)		
Agree	27 (31.0%)	221 (43.1%)		
Strongly agree	38 (43.7%)	217 (42.3%)		

Note: Values are n (%). *p*-values were obtained using Pearson’s chi-square test or the Fisher–Freeman–Halton exact test with 100,000 Monte Carlo samples, as appropriate. q-values are Benjamini–Hochberg-adjusted within the seven counselling items; q < 0.05 was used for inference. Bias-corrected Cramér’s V values are provided in Supplementary File S3, Table S3.

**Table 3:** Frequency of extended health-service provision according to educational qualification.

Extended Health Service	PharmD (n = 87)	B.Pharm (n = 513)	*p*-Value	q-Value
**Vaccination administration**			0.926	0.999
Always	2 (2.3%)	11 (2.1%)		
Mostly	2 (2.3%)	21 (4.1%)		
Sometimes	8 (9.2%)	51 (9.9%)		
Occasionally	15 (17.2%)	95 (18.5%)		
Never	60 (69.0%)	335 (65.3%)		
**Body mass index assessment**			0.053	0.234
Always	6 (6.9%)	27 (5.3%)		
Mostly	9 (10.3%)	33 (6.4%)		
Sometimes	9 (10.3%)	107 (20.9%)		
Occasionally	15 (17.2%)	119 (23.2%)		
Never	48 (55.2%)	227 (44.2%)		
**Blood cholesterol screening**			0.978	0.999
Always	3 (3.4%)	19 (3.7%)		
Mostly	4 (4.6%)	23 (4.5%)		
Sometimes	10 (11.5%)	62 (12.1%)		
Occasionally	16 (18.4%)	80 (15.6%)		
Never	54 (62.1%)	329 (64.1%)		
**Blood glucose screening**			0.536	0.986
Always	5 (5.7%)	28 (5.5%)		
Mostly	3 (3.4%)	27 (5.3%)		
Sometimes	17 (19.5%)	73 (14.2%)		
Occasionally	10 (11.5%)	85 (16.6%)		
Never	52 (59.8%)	300 (58.5%)		
**Blood pressure assessment**			0.999	0.999
Always	5 (5.7%)	28 (5.5%)		
Mostly	5 (5.7%)	32 (6.2%)		
Sometimes	13 (14.9%)	73 (14.2%)		
Occasionally	14 (16.1%)	85 (16.6%)		
Never	50 (57.5%)	295 (57.5%)		
**Smoking-cessation counselling**			0.616	0.986
Always	8 (9.2%)	44 (8.6%)		
Mostly	13 (14.9%)	51 (9.9%)		
Sometimes	21 (24.1%)	114 (22.2%)		
Occasionally	24 (27.6%)	167 (32.6%)		
Never	21 (24.1%)	137 (26.7%)		
**Emergency contraception service**			0.316	0.843
Always	8 (9.2%)	32 (6.2%)		
Mostly	14 (16.1%)	59 (11.5%)		
Sometimes	28 (32.2%)	144 (28.1%)		
Occasionally	21 (24.1%)	166 (32.4%)		
Never	16 (18.4%)	112 (21.8%)		
**Advice on herbal medicines**			0.059	0.234
Always	18 (20.7%)	86 (16.8%)		
Mostly	17 (19.5%)	88 (17.2%)		
Sometimes	23 (26.4%)	132 (25.7%)		
Occasionally	13 (14.9%)	148 (28.8%)		
Never	16 (18.4%)	59 (11.5%)		

Note: Values are n (%). *p*-values were obtained using Pearson’s chi-square test or the Fisher–Freeman–Halton exact test with 100,000 Monte Carlo samples, as appropriate. q-values are Benjamini–Hochberg-adjusted within the eight extended-service items; q < 0.05 was used for inference. Bias-corrected Cramér’s V values are provided in Supplementary File S3, Table S3.

**Table 4:** Perceived barriers to community pharmacy service provision according to educational qualification.

Perceived Barrier	PharmD (n = 87)	B.Pharm (n = 513)	*p*-Value	q-Value
**Insufficient** ** public awareness that such services are available at your community pharmacy.**			0.181	0.214
Strongly disagree	2 (2.3%)	10 (1.9%)		
Disagree	3 (3.4%)	31 (6.0%)		
Neutral	18 (20.7%)	138 (26.9%)		
Agree	37 (42.5%)	231 (45.0%)		
Strongly agree	27 (31.0%)	103 (20.1%)		
**Patients or customers have insufficient time.**			0.139	0.201
Strongly disagree	2 (2.3%)	10 (1.9%)		
Disagree	3 (3.4%)	24 (4.7%)		
Neutral	19 (21.8%)	155 (30.2%)		
Agree	37 (42.5%)	229 (44.6%)		
Strongly agree	26 (29.9%)	95 (18.5%)		
**Insufficient space in the pharmacy premises to protect patient privacy.**			0.162	0.210
Strongly disagree	4 (4.6%)	22 (4.3%)		
Disagree	7 (8.0%)	53 (10.3%)		
Neutral	21 (24.1%)	154 (30.0%)		
Agree	32 (36.8%)	204 (39.8%)		
Strongly agree	23 (26.4%)	80 (15.6%)		
**Insufficient patient demand for these services.**			0.032	0.084
Strongly disagree	3 (3.4%)	13 (2.5%)		
Disagree	8 (9.2%)	54 (10.5%)		
Neutral	25 (28.7%)	183 (35.7%)		
Agree	28 (32.2%)	195 (38.0%)		
Strongly agree	23 (26.4%)	68 (13.3%)		
**Insufficient material resources**			0.032	0.084
Strongly disagree	9 (10.3%)	40 (7.8%)		
Disagree	6 (6.9%)	89 (17.3%)		
Neutral	22 (25.3%)	147 (28.7%)		
Agree	32 (36.8%)	176 (34.3%)		
Strongly agree	18 (20.7%)	61 (11.9%)		
**Insufficient coordination with other healthcare professionals.**			0.010	0.045
Strongly disagree	6 (6.9%)	26 (5.1%)		
Disagree	5 (5.7%)	67 (13.1%)		
Neutral	30 (34.5%)	167 (32.6%)		
Agree	23 (26.4%)	182 (35.5%)		
Strongly agree	23 (26.4%)	71 (13.8%)		
**Lack of access to patient medical records.**			<0.001	<0.001
Strongly disagree	7 (8.0%)	15 (2.9%)		
Disagree	0 (0.0%)	53 (10.3%)		
Neutral	20 (23.0%)	155 (30.2%)		
Agree	34 (39.1%)	217 (42.3%)		
Strongly agree	26 (29.9%)	73 (14.2%)		
**Insufficient integration of community pharmacy with the primary healthcare system.**			0.087	0.142
Strongly disagree	6 (6.9%)	17 (3.3%)		
Disagree	7 (8.0%)	51 (9.9%)		
Neutral	25 (28.7%)	166 (32.4%)		
Agree	27 (31.0%)	198 (38.6%)		
Strongly agree	22 (25.3%)	81 (15.8%)		
**Insufficient pharmacist time.**			0.043	0.093
Strongly disagree	4 (4.6%)	26 (5.1%)		
Disagree	6 (6.9%)	67 (13.1%)		
Neutral	24 (27.6%)	164 (32.0%)		
Agree	29 (33.3%)	179 (34.9%)		
Strongly agree	24 (27.6%)	77 (15.0%)		
**Shortage of pharmacy assistants or technicians.**			0.053	0.098
Strongly disagree	4 (4.6%)	22 (4.3%)		
Disagree	10 (11.5%)	71 (13.8%)		
Neutral	22 (25.3%)	164 (32.0%)		
Agree	27 (31.0%)	181 (35.3%)		
Strongly agree	24 (27.6%)	75 (14.6%)		
**Insufficient confidence in the practical skills required to provide these services.**			0.264	0.286
Strongly disagree	11 (12.6%)	62 (12.1%)		
Disagree	9 (10.3%)	93 (18.1%)		
Neutral	24 (27.6%)	144 (28.1%)		
Agree	28 (32.2%)	158 (30.8%)		
Strongly agree	15 (17.2%)	56 (10.9%)		
**Insufficient confidence in the knowledge required to interpret and discuss these services with patients.**			0.498	0.498
Strongly disagree	10 (11.5%)	52 (10.1%)		
Disagree	10 (11.5%)	91 (17.7%)		
Neutral	25 (28.7%)	153 (29.8%)		
Agree	29 (33.3%)	164 (32.0%)		
Strongly agree	13 (14.9%)	53 (10.3%)		
**Insufficient training to provide these services.**			0.006	0.037
Strongly disagree	5 (5.7%)	48 (9.4%)		
Disagree	8 (9.2%)	88 (17.2%)		
Neutral	21 (24.1%)	149 (29.0%)		
Agree	34 (39.1%)	178 (34.7%)		
Strongly agree	19 (21.8%)	50 (9.7%)		

Note: Values are n (%). *p*-values were obtained using Pearson’s chi-square test or the Fisher–Freeman–Halton exact test with 100,000 Monte Carlo samples, as appropriate. q-values are Benjamini–Hochberg-adjusted within the thirteen barrier items; q < 0.05 was used for inference. Bias-corrected Cramér’s V values are provided in Supplementary File S3, Table S3.

## Data Availability

The de-identified data supporting the findings of this study are available from the corresponding author upon reasonable request and subject to institutional and ethical approval. The data are not publicly available because unrestricted public sharing was not specified in the original ethics approval and participant information.

## Supplementary Materials

The following supporting information can be downloaded at: https://www.mdpi.com/article/10.3390/healthcare14203410/s1, Supplementary File S1: Study questionnaire items and response options; Supplementary File S2: STROBE Reporting Checklist for a Cross-Sectional Study; Supplementary File S3: Statistical Tables; Supplementary File S4: Consent statement and participant-facing survey.

## References

[1] Alhazmi M., Bajuayfir A., Cheema E., Elrggal M., Ali M. (2022). Evaluation of Current Community Pharmacist Practice in Saudi Arabia—A Cross-Sectional Study from Pharmacists’ Perspective (Part II). Pharmacy.

[2] DiPietro Mager N., Bright D. (2023). Advancing Public Health through Community Pharmacy Practice. Pharmacy.

[3] Milosavljevic A., Aspden T., Harrison J. (2018). Community pharmacist-led interventions and their impact on patients’ medication adherence and other health outcomes: A systematic review. Int. J. Pharm. Pract..

[4] Valliant S.N., Burbage S.C., Pathak S., Urick B.Y. (2022). Pharmacists as Accessible Health Care Providers: Quantifying the Opportunity. J. Manag. Care Spec. Pharm..

[5] Yuan C., Ding Y., Zhou K., Huang Y., Xi X. (2019). Clinical Outcomes of Community Pharmacy Services: A Systematic Review and Meta-Analysis. Health Soc. Care Community.

[6] Buss V.H., Shield A., Kosari S., Naunton M. (2018). The Impact of Clinical Services Provided by Community Pharmacies on the Australian Healthcare System: A Review of the Literature. J. Pharm. Policy Pract..

[7] Jackson E., McGrath S.H., Carroll J.C., McGivney M.S., Pitzarella S., Coley K.C. (2022). Implementation of a Health Risk Assessment into Workflow of the Appointment-Based Model at an Independent Community Pharmacy. Pharmacy.

[8] Loh P., Chua S.S., Karuppannan M. (2021). The Extent and Barriers in Providing Pharmaceutical Care Services by Community Pharmacists in Malaysia: A Cross-Sectional Study. BMC Health Serv. Res..

[9] Agomo C.O., Ogunleye J., Portlock J. (2020). Enhancing the Public Health Role of Community Pharmacists—A Qualitative Research Utilising the Theoretical Domains Framework. Innov. Pharm..

[10] Kroenert A.C., Bertsche T. (2024). Implementation, Barriers, Solving Strategies and Future Perspectives of Reimbursed Community Pharmacy Services—A Nationwide Survey for Community Pharmacies in Germany. BMC Health Serv. Res..

[11] Karia A.M., Chen L., Norman R., Robinson S., Webster Griffiths R., Sim T.F. (2025). Barriers and Facilitators Affecting the Implementation of Community Pharmacist-Led Professional Services in Organisation for Economic Co-operation and Development Member Countries: A Scoping Review in the Context of Expanded Scope. Int. J. Pharm. Pract..

[12] Khayyat S.M., Haseeb A., Alkaabi Z.H., Bahaziq A.M., Alhomayani A.M., Alhifany A.A., Alrammaal H.H., Jaha A.Y. (2024). Clinical Services, Barriers, and Public Perspectives of Community Pharmacies in Saudi Arabia: A Cross-Sectional Study. J. Patient Exp..

[13] Alghamdi K.S., Petzold M., Alsugoor M.H., Makeen H.A., Persson C.L., Hussain-Alkhateeb L. (2025). Multi-Stakeholder Perspective on Community Pharmacy Services in Saudi Arabia: A Systematic Review and Meta-Analyses for 2010–2020. Explor. Res. Clin. Soc. Pharm..

[14] Alzahrani F., Khairi N.B., Alattas B.O., Alrehaili T.H., Aljehani G.S., Alahmadi R.B., Refi D.M., Aljohani L.S., Fadil H.A., Alnezary F.S. (2025). Community Pharmacists’ Readiness for Minor Ailment Services in Saudi Arabia: A Cross-Sectional Study of Perceptions, Barriers, and Facilitators. Int. J. Pharm. Pract..

[15] Alghamdi K.S., Alsugoor M.H., Petzold M., Hussain-Alkhateeb L., Ljungberg Persson C. (2025). Multi-Stakeholder Perspectives of Community Pharmacies’ Extended Services in Al Baha Province, Saudi Arabia: A Qualitative Study. Glob. Public Health.

[16] Alharbi R.T., Alhazmi R.H., Alotaibi S.M., Adawi L.A., Alfaify M.J., Jali A.A., Izzuldeen R.T., Alraythi S.J., Someli K.H., Jerebi K.J. (2026). A Multidimensional Analysis of the Determinants of Medication Counseling Behaviors: A Cross-Sectional Study of Community Pharmacists in Jazan, Saudi Arabia. Cureus.

[17] Salem H.H., Siddiqua A., Aljali R.S., Alshardi A.I., Abusllam R.M., Alqahtani R.M., Alshehri L.S., Bazan N.S. (2026). Knowledge, Practice and Barriers of Community Pharmacists towards Asthma Management: A Cross-Sectional Study in Saudi Arabia. Healthcare.

[18] Alfarhan M., Haqwi M.F., Musayyikh A.H., Ashqar J., Suwidi L.Y., Fageh A.H., Alajam E.A., Almansour H., Alshammari T.M., Al-Qahtani S. (2026). Drug–Drug Interaction Knowledge, Practices, and Barriers in Community Pharmacies: A Cross-Sectional Study from Jazan Region, Saudi Arabia. Pharmacy.

[19] Korayem G.B., Badreldin H.A., Eljaaly K., Aldemerdash A., Al-Suhaibani L.K., Joharji H., Aljuhani O., Al-Omari B.A., Almudaiheem H.Y., Alhifany A.A. (2021). Clinical Pharmacy Definition, Required Education, Training and Practice in Saudi Arabia: A Position Statement by the Saudi Society of Clinical Pharmacy. Saudi Pharm. J..

[20] Alfaifi S., Bridges S., Arakawa N. (2026). Mapping Undergraduate Pharmacy Curricula in Saudi Arabia against a National Competency Framework: A Case Study of Two PharmD Programmes. Curr. Pharm. Teach. Learn..

[21] von Elm E., Altman D.G., Egger M., Pocock S.J., Gøtzsche P.C., Vandenbroucke J.P. (2007). The Strengthening the Reporting of Observational Studies in Epidemiology (STROBE) Statement: Guidelines for Reporting Observational Studies. PLoS Med..

[22] Alhazmi A.F., Alghuthmi F.A., Hamoh M.A., Almasari K.A., Zagzoug A.M., Kenkar M.M., Tayeb S.M., Aldurdunji M.M., Alsheikh E.O., Almuzaini O.K. (2026). Evaluation of Community Pharmacists’ Knowledge and Real-World Practices in Drug Interaction Management: A Cross-Sectional Study. Front. Med..

[23] Noorsaeed S.M.W., Alshammari H., Weir N., Kurdi A. (2025). Exploring Community Pharmacy Services in Gulf Cooperation Council Countries: A Scoping Review. Int. J. Pharm. Pract..

[24] Alshahrani S.M. (2026). A Mixed-Methods SWOT Analysis of Community Pharmacy Services in Saudi Arabia: Stakeholder Perspectives and Strategic Framework. Front. Med..

[25] Khobrani M. (2026). Knowledge, Attitudes, and Practices of Community Pharmacists Regarding the Management of Diabetes During Ramadan in the Aseer Region, Saudi Arabia. Front. Pharmacol..

[26] Alsharif S.T. (2026). Community Pharmacy Staff Preparedness and Challenges in Counseling Cancer Patients and Survivors: A Study from Saudi Arabia. BMC Health Serv. Res..

[27] Algarni M., Alghamdi S., Barakat B.M., Alshammari T.M., Alzahrani A., Alzahrani A., Sumayli H., Al-Qahtani S.A., Mantargi M.J.S. (2026). Evaluating Community Pharmacists’ Practices in Managing Insomnia in Saudi Arabia: A Cross-Sectional Study Using Simulated Patient Visits. Front. Public Health.

