# Supplementary material for: Self-Reported Provision of Community Pharmacy Services and Perceived Barriers in Saudi Arabia: A Cross-Sectional Survey
Source: Healthcare (Basel). 2026 Oct 13;14(20):3410. doi: 10.3390/healthcare14203410 (PMC13704151; doi:10.3390/healthcare14203410)
Supplement: Supplementary file 1 [file healthcare-14-03410-s001.zip › Supplementary File S1 - Questionnaire.pdf]

Supplementary File S1. Study Questionnaire Items and Response Options

Self-Reported Provision of Community Pharmacy Services and Perceived Barriers in Saudi Arabia: A Cross-Sectional Survey

Note: The questionnaire items and response categories below present the content of the survey administered in this study. The consent statement and participant-facing survey layout are provided in Supplementary File S4.

Section S1. Demographic and Professional Characteristics

No.	Questionnaire item	Response options
1	Gender	Male; Female
2	Age (years)	20–25; 26–30; 31–35; 36–40; More than 40 years
3	Years of community pharmacy practice experience	1–5; 6–10; 11–15; More than 16 years
4	Average daily customer count	1–50; 51–100; More than 100 customers
5	Nationality	Saudi; Non-Saudi
6	Educational background	Bachelor of Pharmacy (B.Pharm); Doctor of Pharmacy (PharmD)
7	Location of your current work	Inside Riyadh; Outside Riyadh
8	If inside Riyadh, what is the location of your pharmacy?	West; North; East; South
9	If outside Riyadh, what is the location of your pharmacy?	West; North; East; South
10	Type of community pharmacy	Chain; Independent

Section S2A. Medication-Counselling Practices

Instruction: Please indicate your level of agreement with each statement.

Response options: Strongly agree; Agree; Neither agree nor disagree; Disagree; Strongly disagree

No.	Questionnaire item	Response options
1	I provide patient counselling with every dispensing.	Agreement scale
2	I provide patients with instructions on drug administration, dosage, side effects, and precautions.	Agreement scale
3	I detect drug–drug interactions and adverse drug reactions during counselling.	Agreement scale
4	I assist in identifying and reporting medication errors to the appropriate authority.	Agreement scale
5	I provide advice on drug therapy for chronic non-communicable diseases.	Agreement scale
6	I provide counselling on non-prescription options for the common cold or influenza.	Agreement scale
7	I provide patients with medical information about diseases and medications.	Agreement scale

Section S2B. Extended Health Services

Instruction: How frequently do you provide each of the following services?

Response options: Never; Occasionally; Sometimes; Mostly; Always

No.	Questionnaire item	Response options
1	Vaccination administration	Frequency scale
2	Body mass index assessment	Frequency scale
3	Blood glucose screening	Frequency scale
4	Blood pressure assessment	Frequency scale
5	Blood cholesterol screening	Frequency scale
6	Smoking-cessation counselling	Frequency scale
7	Emergency contraception service	Frequency scale
8	Advice on herbal medicines	Frequency scale

Section S3. Perceived Barriers to Service Provision

Response options: Strongly agree; Agree; Neutral; Disagree; Strongly disagree

No.	Questionnaire item	Response options
1	Insufficient public awareness that such services are available at your community pharmacy.	Agreement scale
2	Patients or customers have insufficient time.	Agreement scale
3	Insufficient space in the pharmacy premises to protect patient privacy.	Agreement scale
4	Insufficient patient demand for these services.	Agreement scale
5	Insufficient material resources, such as computers, internet access, pharmacy software, glucose-testing supplies, and needle-disposal procedures.	Agreement scale
6	Insufficient coordination with other healthcare professionals.	Agreement scale
7	Lack of access to patient medical records.	Agreement scale
8	Insufficient integration of community pharmacy with the primary healthcare system.	Agreement scale
9	Insufficient pharmacist time.	Agreement scale
10	Shortage of pharmacy assistants or technicians.	Agreement scale
11	Insufficient confidence in the practical skills required to provide these services.	Agreement scale
12	Insufficient confidence in the knowledge required to interpret and discuss these services with patients.	Agreement scale
13	Insufficient training to provide these services.	Agreement scale
