# Supplementary material for: Self-Reported Provision of Community Pharmacy Services and Perceived Barriers in Saudi Arabia: A Cross-Sectional Survey
Source: Healthcare (Basel). 2026 Oct 13;14(20):3410. doi: 10.3390/healthcare14203410 (PMC13704151; doi:10.3390/healthcare14203410)
Supplement: Supplementary file 1 [file healthcare-14-03410-s001.zip › Supplementary File S2 - STROBE Checklist.pdf]

Supplementary File S2.

STROBE Reporting Checklist for a Cross-Sectional Study

Manuscript: *Self-Reported Provision of Community Pharmacy Services and Perceived Barriers in Saudi Arabia: A Cross-Sectional Survey*

Page numbers refer to the submitted manuscript PDF.

Item	Reporting topic	Location in manuscript
1a	Title and study design	Title, page 1
1b	Informative abstract	Abstract, page 1
2	Background and rationale	Introduction, pages 1–2
3	Objectives	Introduction, page 2
4	Study design	Section 2.1, page 3
5	Setting, location, and dates	Section 2.1, page 3
6	Participant eligibility and selection	Sections 2.1 and 2.4, pages 3–4
7	Variables and outcomes	Sections 2.2 and 2.6, pages 3–5
8	Data sources and measurement	Sections 2.2–2.3, pages 3–4; Supplementary Files S1 and S4
9	Potential sources of bias	Section 4.5, pages 17–18
10	Study-size calculation	Section 2.4, page 4
11	Handling of quantitative variables	Section 2.6, page 5
12a	Statistical methods and confounding control	Section 2.6, pages 4–5; Supplementary File S3 (Tables S1–S5)
12b	Subgroup and other analyses	Education-group comparisons, adjusted threshold-specific models, post hoc sensitivity analyses, and exploratory domain-level analyses, Sections 2.6 and 3.2–3.5, pages 4–14
12c	Missing-data approach	Primary 28 questionnaire items: complete data for all 600 respondents, Section 3.1, page 5. Geographic sensitivity analysis: unusable directional data were excluded, Section 2.6, page 5; Section 3.3, page 10
12d	Sampling strategy in analysis	Not applicable to probability-sample weighting; recruitment was multichannel non-probability convenience sampling without a national sampling frame (Section 2.1, page 3)
12e	Sensitivity analyses	Post hoc Firth and geographic sensitivity analyses, Section 2.6, page 5 and Section 3.3, pages 10–11; Supplementary File S3, Tables S5A–S5B
13a	Numbers at study stages	Final analytic sample: Section 2.4, page 4 and Section 3.1, page 5; invitation denominator/other recruitment-stage counts were not documented (Section 2.1, page 3)
13b	Reasons for non-participation	Invitation denominator and reasons for non-participation were not documented; Section 2.1, page 3
13c	Participant flow diagram	Not available because the invitation denominator was not documented (Section 2.1, page 3)
14a	Participant characteristics	Section 3.1 and Table 1, pages 5–6
14b	Missing data for variables	No item-level missing data for the 28 questionnaire items: Section 3.1, page 5; directional sensitivity-analysis data: Section 3.3, page 10
14c	Follow-up time	Not applicable to a cross-sectional survey
15	Outcome and exposure data	Sections 3.2–3.4 and Tables 2–4, pages 6–13
16a	Unadjusted and adjusted estimates	Sections 3.2–3.5, pages 6–14; Supplementary File S3, Tables S1, S3, S4, and S5A–S5B
16b	Category boundaries	Section 2.6, pages 4–5; Tables 1–4, pages 6–13
16c	Absolute-risk translation	Not applicable; no risk estimates were calculated
17	Other and sensitivity analyses	Post hoc Firth and geographic sensitivity analyses: Section 2.6, page 5 and Section 3.3, pages 10–11; Supplementary File S3, Tables S5A–S5B. Exploratory domain-level analyses: Section 3.5, pages 13–14; Supplementary File S3, Table S4
18	Key results	Section 4.1, page 14
19	Limitations	Section 4.5, pages 17–18
20	Interpretation	Sections 4.1–4.4, pages 14–17
21	Generalisability	Section 4.5, pages 17–18; Conclusion, page 18
22	Funding and role of funders	Funding, page 19
