# Supplementary material for: Self-Reported Provision of Community Pharmacy Services and Perceived Barriers in Saudi Arabia: A Cross-Sectional Survey
Source: Healthcare (Basel). 2026 Oct 13;14(20):3410. doi: 10.3390/healthcare14203410 (PMC13704151; doi:10.3390/healthcare14203410)
Supplement: Supplementary file 1 [file healthcare-14-03410-s001.zip › Supplementary File S3 - Statistical Tables.pdf]

Supplementary File S3. Statistical Tables

Self-Reported Provision of Community Pharmacy Services and Perceived Barriers in Saudi Arabia: A Cross-Sectional Survey

Supplementary Table S1. Full Adjusted Threshold-Specific Logistic Regression Models for Extended-Service Provision

Separate multivariable logistic regression models were fitted for each extended service at two thresholds: (1) any provision (occasionally, sometimes, mostly, or always) versus never and (2) frequent provision (mostly or always) versus less frequent provision. Adjusted odds ratios (aORs) greater than 1 indicate higher odds of the respective outcome. Models were adjusted for age group, gender, nationality, educational qualification, daily customer volume, location (inside versus outside Riyadh), and pharmacy type. Benjamini–Hochberg false discovery rate (FDR) correction was applied separately within each threshold family (88 predictor tests per family).

Vaccination administration

Any provision vs never (events = 205 of 600)

Predictor	aOR	95% CI lower	95% CI upper	p-value	q-value
Age 26–30 vs 20–25	2.94	1.17	7.42	0.022	0.116
Age 31–35 vs 20–25	3.13	1.18	8.31	0.022	0.116
Age 36–40 vs 20–25	2.84	1.02	7.89	0.045	0.173
Age >40 vs 20–25	1.43	0.48	4.29	0.522	0.851
Female vs Male	0.85	0.45	1.61	0.609	0.879
Non-Saudi vs Saudi	1.24	0.68	2.26	0.491	0.815
B.Pharm vs PharmD	0.75	0.40	1.41	0.366	0.700
Customers 51–100 vs 1–50	0.97	0.58	1.62	0.903	0.922
Customers >100 vs 1–50	1.32	0.77	2.29	0.316	0.672
Outside vs Inside Riyadh	2.40	1.65	3.48	<0.001	<0.001
Chain vs Independent	1.96	1.06	3.64	0.032	0.155

Frequent provision vs less frequent (events = 36 of 600)

Predictor	aOR	95% CI lower	95% CI upper	p-value	q-value
Age 26–30 vs 20–25	2.15	0.23	19.70	0.498	0.859
Age 31–35 vs 20–25	1.88	0.18	19.06	0.594	0.918
Age 36–40 vs 20–25	3.70	0.36	38.13	0.272	0.683
Age >40 vs 20–25	1.31	0.10	16.56	0.834	0.983
Female vs Male	1.90	0.51	7.09	0.340	0.731
Non-Saudi vs Saudi	1.54	0.38	6.33	0.546	0.904
B.Pharm vs PharmD	0.92	0.22	3.74	0.904	0.983
Customers 51–100 vs 1–50	2.05	0.55	7.73	0.288	0.702
Customers >100 vs 1–50	2.73	0.72	10.39	0.140	0.474
Outside vs Inside Riyadh	1.90	0.93	3.91	0.080	0.353
Chain vs Independent	NE*	—	—	0.919	0.983

Body mass index assessment

Any provision vs never (events = 325 of 600)

Predictor	aOR	95% CI lower	95% CI upper	p-value	q-value
Age 26–30 vs 20–25	1.57	0.75	3.31	0.235	0.560
Age 31–35 vs 20–25	1.90	0.85	4.26	0.120	0.351
Age 36–40 vs 20–25	1.15	0.49	2.73	0.745	0.902
Age >40 vs 20–25	0.86	0.34	2.16	0.747	0.902
Female vs Male	0.62	0.35	1.13	0.120	0.351
Non-Saudi vs Saudi	1.57	0.89	2.76	0.118	0.351
B.Pharm vs PharmD	0.91	0.50	1.65	0.760	0.902
Customers 51–100 vs 1–50	0.94	0.58	1.52	0.811	0.902
Customers >100 vs 1–50	1.45	0.85	2.47	0.171	0.443
Outside vs Inside Riyadh	3.71	2.52	5.46	<0.001	<0.001
Chain vs Independent	2.11	1.23	3.62	0.007	0.058

Frequent provision vs less frequent (events = 75 of 600)

Predictor	aOR	95% CI lower	95% CI upper	p-value	q-value
Age 26–30 vs 20–25	2.43	0.63	9.31	0.195	0.554
Age 31–35 vs 20–25	1.44	0.34	6.12	0.620	0.920
Age 36–40 vs 20–25	3.61	0.83	15.59	0.086	0.360
Age >40 vs 20–25	1.73	0.36	8.36	0.493	0.859
Female vs Male	0.70	0.28	1.79	0.459	0.859
Non-Saudi vs Saudi	1.13	0.44	2.89	0.795	0.983
B.Pharm vs PharmD	0.35	0.14	0.85	0.021	0.172
Customers 51–100 vs 1–50	1.22	0.53	2.81	0.645	0.920
Customers >100 vs 1–50	2.41	1.04	5.60	0.041	0.197
Outside vs Inside Riyadh	2.07	1.22	3.52	0.007	0.122
Chain vs Independent	5.49	1.26	23.91	0.023	0.172

Blood cholesterol screening

Any provision vs never (events = 217 of 600)

Predictor	aOR	95% CI lower	95% CI upper	p-value	q-value
Age 26–30 vs 20–25	2.49	1.06	5.81	0.035	0.164
Age 31–35 vs 20–25	2.33	0.94	5.78	0.068	0.229
Age 36–40 vs 20–25	2.07	0.79	5.42	0.137	0.364
Age >40 vs 20–25	1.09	0.39	3.07	0.871	0.922
Female vs Male	1.08	0.57	2.02	0.821	0.902
Non-Saudi vs Saudi	0.96	0.52	1.75	0.882	0.922
B.Pharm vs PharmD	0.72	0.39	1.34	0.300	0.672
Customers 51–100 vs 1–50	1.18	0.69	2.00	0.544	0.871
Customers >100 vs 1–50	2.11	1.21	3.69	0.009	0.058
Outside vs Inside Riyadh	2.78	1.92	4.03	<0.001	<0.001
Chain vs Independent	3.06	1.55	6.06	0.001	0.017

Frequent provision vs less frequent (events = 49 of 600)

Predictor	aOR	95% CI lower	95% CI upper	p-value	q-value
Age 26–30 vs 20–25	1.57	0.30	8.14	0.593	0.918
Age 31–35 vs 20–25	1.17	0.20	6.80	0.863	0.983
Age 36–40 vs 20–25	2.35	0.39	14.05	0.350	0.733
Age >40 vs 20–25	1.14	0.17	7.83	0.895	0.983
Female vs Male	2.11	0.70	6.38	0.186	0.546
Non-Saudi vs Saudi	1.51	0.46	4.93	0.498	0.859
B.Pharm vs PharmD	0.80	0.25	2.52	0.703	0.951
Customers 51–100 vs 1–50	3.78	1.05	13.67	0.043	0.197
Customers >100 vs 1–50	3.91	1.05	14.58	0.042	0.197
Outside vs Inside Riyadh	2.07	1.10	3.90	0.023	0.172
Chain vs Independent	NE*	—	—	0.943	0.983

Blood glucose screening

Any provision vs never (events = 248 of 600)

Predictor	aOR	95% CI lower	95% CI upper	p-value	q-value
Age 26–30 vs 20–25	2.22	0.97	5.08	0.058	0.205
Age 31–35 vs 20–25	2.52	1.04	6.10	0.041	0.172
Age 36–40 vs 20–25	1.44	0.56	3.69	0.451	0.778
Age >40 vs 20–25	0.88	0.32	2.43	0.802	0.902
Female vs Male	0.85	0.45	1.58	0.607	0.879
Non-Saudi vs Saudi	1.03	0.57	1.87	0.912	0.922
B.Pharm vs PharmD	0.79	0.42	1.46	0.444	0.778
Customers 51–100 vs 1–50	1.27	0.76	2.12	0.356	0.700
Customers >100 vs 1–50	1.92	1.11	3.32	0.020	0.116
Outside vs Inside Riyadh	3.71	2.56	5.38	<0.001	<0.001
Chain vs Independent	1.87	1.03	3.40	0.039	0.172

Frequent provision vs less frequent (events = 63 of 600)

Predictor	aOR	95% CI lower	95% CI upper	p-value	q-value
Age 26–30 vs 20–25	0.79	0.22	2.85	0.715	0.953
Age 31–35 vs 20–25	0.65	0.16	2.64	0.551	0.904
Age 36–40 vs 20–25	1.03	0.24	4.34	0.970	0.983
Age >40 vs 20–25	0.89	0.20	3.99	0.877	0.983
Female vs Male	1.68	0.61	4.62	0.315	0.712
Non-Saudi vs Saudi	1.53	0.53	4.38	0.430	0.840
B.Pharm vs PharmD	0.93	0.33	2.63	0.895	0.983
Customers 51–100 vs 1–50	2.99	1.05	8.51	0.041	0.197
Customers >100 vs 1–50	3.16	1.08	9.28	0.036	0.197
Outside vs Inside Riyadh	1.86	1.07	3.25	0.029	0.192
Chain vs Independent	NE*	—	—	0.963	0.983

Blood pressure assessment

Any provision vs never (events = 255 of 600)

Predictor	aOR	95% CI lower	95% CI upper	p-value	q-value
Age 26–30 vs 20–25	1.22	0.55	2.73	0.619	0.879
Age 31–35 vs 20–25	1.76	0.75	4.14	0.197	0.493
Age 36–40 vs 20–25	1.17	0.47	2.92	0.736	0.902
Age >40 vs 20–25	0.82	0.31	2.17	0.690	0.902
Female vs Male	1.16	0.62	2.14	0.645	0.902
Non-Saudi vs Saudi	1.27	0.70	2.30	0.433	0.778
B.Pharm vs PharmD	0.70	0.38	1.29	0.257	0.595
Customers 51–100 vs 1–50	1.94	1.15	3.27	0.013	0.082
Customers >100 vs 1–50	2.42	1.38	4.23	0.002	0.022
Outside vs Inside Riyadh	3.43	2.37	4.96	<0.001	<0.001
Chain vs Independent	2.61	1.38	4.93	0.003	0.030

Frequent provision vs less frequent (events = 70 of 600)

Predictor	aOR	95% CI lower	95% CI upper	p-value	q-value
Age 26–30 vs 20–25	1.11	0.32	3.84	0.873	0.983
Age 31–35 vs 20–25	0.82	0.21	3.15	0.774	0.983
Age 36–40 vs 20–25	1.08	0.27	4.42	0.910	0.983
Age >40 vs 20–25	0.83	0.19	3.66	0.804	0.983
Female vs Male	1.40	0.55	3.56	0.480	0.859
Non-Saudi vs Saudi	1.25	0.48	3.23	0.648	0.920
B.Pharm vs PharmD	0.85	0.33	2.18	0.740	0.972
Customers 51–100 vs 1–50	3.43	1.23	9.53	0.018	0.172
Customers >100 vs 1–50	4.12	1.44	11.79	0.008	0.123
Outside vs Inside Riyadh	1.56	0.92	2.64	0.097	0.389
Chain vs Independent	9.20	1.23	68.79	0.031	0.192

Smoking-cessation counselling

Any provision vs never (events = 442 of 600)

Predictor	aOR	95% CI lower	95% CI upper	p-value	q-value
Age 26–30 vs 20–25	2.09	0.99	4.40	0.052	0.191
Age 31–35 vs 20–25	1.18	0.53	2.62	0.680	0.902
Age 36–40 vs 20–25	0.86	0.37	2.01	0.735	0.902
Age >40 vs 20–25	0.89	0.36	2.18	0.792	0.902
Female vs Male	0.73	0.40	1.35	0.318	0.672
Non-Saudi vs Saudi	0.96	0.53	1.74	0.894	0.922
B.Pharm vs PharmD	0.76	0.40	1.43	0.389	0.728
Customers 51–100 vs 1–50	1.26	0.77	2.07	0.358	0.700
Customers >100 vs 1–50	2.18	1.22	3.89	0.008	0.058
Outside vs Inside Riyadh	2.02	1.33	3.07	0.001	0.015
Chain vs Independent	0.61	0.34	1.08	0.091	0.298

Frequent provision vs less frequent (events = 116 of 600)

Predictor	aOR	95% CI lower	95% CI upper	p-value	q-value
Age 26–30 vs 20–25	1.21	0.48	3.02	0.682	0.938
Age 31–35 vs 20–25	0.58	0.21	1.61	0.295	0.702
Age 36–40 vs 20–25	0.73	0.25	2.13	0.563	0.904
Age >40 vs 20–25	0.60	0.19	1.88	0.384	0.785
Female vs Male	1.21	0.58	2.52	0.619	0.920
Non-Saudi vs Saudi	1.83	0.86	3.87	0.114	0.411
B.Pharm vs PharmD	0.56	0.27	1.16	0.117	0.411
Customers 51–100 vs 1–50	1.69	0.88	3.25	0.114	0.411
Customers >100 vs 1–50	1.65	0.82	3.31	0.157	0.478
Outside vs Inside Riyadh	1.72	1.11	2.65	0.015	0.166
Chain vs Independent	10.65	2.53	44.88	0.001	0.046

Emergency contraception service

Any provision vs never (events = 472 of 600)

Predictor	aOR	95% CI lower	95% CI upper	p-value	q-value
Age 26–30 vs 20–25	1.52	0.66	3.48	0.321	0.672
Age 31–35 vs 20–25	1.53	0.62	3.74	0.355	0.700
Age 36–40 vs 20–25	0.68	0.27	1.71	0.413	0.757
Age >40 vs 20–25	0.86	0.32	2.29	0.761	0.902
Female vs Male	1.15	0.58	2.29	0.693	0.902
Non-Saudi vs Saudi	0.93	0.48	1.79	0.830	0.902
B.Pharm vs PharmD	0.94	0.47	1.88	0.867	0.922
Customers 51–100 vs 1–50	0.85	0.49	1.47	0.556	0.873
Customers >100 vs 1–50	0.91	0.49	1.69	0.769	0.902
Outside vs Inside Riyadh	1.81	1.17	2.81	0.008	0.058
Chain vs Independent	0.60	0.32	1.16	0.128	0.351

Frequent provision vs less frequent (events = 113 of 600)

Predictor	aOR	95% CI lower	95% CI upper	p-value	q-value
Age 26–30 vs 20–25	0.98	0.42	2.30	0.967	0.983
Age 31–35 vs 20–25	0.82	0.32	2.10	0.682	0.938
Age 36–40 vs 20–25	0.74	0.27	2.06	0.565	0.904
Age >40 vs 20–25	0.94	0.33	2.66	0.900	0.983
Female vs Male	2.98	1.51	5.86	0.002	0.046
Non-Saudi vs Saudi	1.49	0.74	2.99	0.266	0.683
B.Pharm vs PharmD	0.75	0.38	1.48	0.412	0.824
Customers 51–100 vs 1–50	2.31	1.22	4.38	0.010	0.128
Customers >100 vs 1–50	1.56	0.77	3.16	0.213	0.569
Outside vs Inside Riyadh	1.00	0.65	1.56	0.983	0.983
Chain vs Independent	3.72	1.52	9.09	0.004	0.087

Advice on herbal medicines

Any provision vs never (events = 525 of 600)

Predictor	aOR	95% CI lower	95% CI upper	p-value	q-value
Age 26–30 vs 20–25	2.42	1.02	5.72	0.044	0.173
Age 31–35 vs 20–25	2.11	0.81	5.52	0.127	0.351
Age 36–40 vs 20–25	1.44	0.53	3.95	0.476	0.806
Age >40 vs 20–25	2.12	0.67	6.75	0.202	0.493
Female vs Male	0.92	0.44	1.92	0.822	0.902
Non-Saudi vs Saudi	1.23	0.58	2.61	0.581	0.879
B.Pharm vs PharmD	1.22	0.59	2.55	0.590	0.879
Customers 51–100 vs 1–50	1.08	0.56	2.09	0.809	0.902
Customers >100 vs 1–50	1.12	0.53	2.37	0.758	0.902
Outside vs Inside Riyadh	1.15	0.67	1.98	0.614	0.879
Chain vs Independent	0.97	0.48	1.98	0.934	0.934

Frequent provision vs less frequent (events = 209 of 600)

Predictor	aOR	95% CI lower	95% CI upper	p-value	q-value
Age 26–30 vs 20–25	1.04	0.50	2.19	0.913	0.983
Age 31–35 vs 20–25	0.89	0.40	1.99	0.773	0.983
Age 36–40 vs 20–25	1.01	0.43	2.40	0.975	0.983
Age >40 vs 20–25	0.80	0.32	1.99	0.630	0.920
Female vs Male	1.25	0.69	2.30	0.462	0.859
Non-Saudi vs Saudi	1.33	0.74	2.41	0.341	0.731
B.Pharm vs PharmD	0.64	0.35	1.17	0.146	0.474
Customers 51–100 vs 1–50	1.45	0.87	2.42	0.151	0.474
Customers >100 vs 1–50	1.33	0.77	2.30	0.312	0.712
Outside vs Inside Riyadh	1.26	0.88	1.82	0.209	0.569
Chain vs Independent	8.26	3.47	19.66	<0.001	<0.001

NE* = not estimable because quasi-complete separation and extremely sparse threshold cells prevented stable coefficient and confidence-interval estimation. The displayed p- and q-values are retained for completeness within the prespecified multiplicity-adjustment family, but these unstable rows were not used for substantive interpretation.

Supplementary Table S2. Reliability and Item Diagnostics

Cronbach's alpha was calculated separately for the counselling, extended-services, and perceived-barriers domains. Bootstrap 95% confidence intervals were estimated from 2,000 resamples. The 50 pilot participants were independent of and excluded from the final analytical sample of 600.

Sample	N	Domain	Items	Cronbach α	Standardised α	Bootstrap 95% CI lower	Bootstrap 95% CI upper
Pilot	50	Counselling	7	0.917	0.921	0.854	0.952
Pilot	50	Extended services	8	0.860	0.864	0.723	0.923
Pilot	50	Barriers	13	0.865	0.865	0.736	0.923
Main sample	600	Counselling	7	0.930	0.930	0.915	0.942
Main sample	600	Extended services	8	0.886	0.889	0.865	0.903
Main sample	600	Barriers	13	0.932	0.932	0.920	0.942

Main-sample item diagnostics

Domain	Item	Mean	SD	Corrected item–total correlation	Alpha if item deleted	Effect of item deletion
Counselling	Patient counselling with every dispensing	4.342	0.789	0.713	0.925	No material improvement in α
Counselling	Instructions on drug administration, dosage, side effects, and precautions	4.272	0.848	0.792	0.918	No material improvement in α
Counselling	Detection of drug–drug interactions and adverse drug reactions	4.127	0.890	0.795	0.917	No material improvement in α
Counselling	Identification and reporting of medication errors	4.077	0.945	0.768	0.920	No material improvement in α
Counselling	Advice on drug therapy for chronic non-communicable diseases	4.185	0.882	0.834	0.913	No material improvement in α
Counselling	Counselling on non-prescription options for the common cold or influenza	4.253	0.878	0.770	0.920	No material improvement in α
Counselling	Medical information about diseases and medications	4.180	0.921	0.765	0.920	No material improvement in α
Extended services	Vaccination administration	1.582	0.965	0.700	0.870	No material improvement in α
Extended services	Body mass index assessment	2.040	1.195	0.642	0.873	No material improvement in α
Extended services	Blood cholesterol screening	1.682	1.081	0.752	0.863	No material improvement in α
Extended services	Blood glucose screening	1.828	1.185	0.800	0.857	No material improvement in α
Extended services	Blood pressure assessment	1.857	1.201	0.795	0.857	No material improvement in α

Domain	Item	Mean	SD	Corrected item–total correlation	Alpha if item deleted	Effect of item deletion
Extended services	Smoking-cessation counselling	2.435	1.228	0.636	0.874	No material improvement in α
Extended services	Emergency contraception service	2.517	1.150	0.493	0.888	No material improvement in α
Extended services	Advice on herbal medicines	3.003	1.280	0.486	0.890	No material improvement in α
Barriers	Insufficient public awareness of available services	3.783	0.915	0.580	0.931	No material improvement in α
Barriers	Patients or customers have insufficient time	3.762	0.892	0.537	0.932	No material improvement in α
Barriers	Insufficient space to protect patient privacy	3.550	1.026	0.584	0.931	No material improvement in α
Barriers	Insufficient patient demand	3.518	0.960	0.654	0.928	No material improvement in α
Barriers	Insufficient material resources	3.288	1.130	0.731	0.926	No material improvement in α
Barriers	Insufficient coordination with other healthcare professionals	3.428	1.058	0.757	0.925	No material improvement in α
Barriers	Lack of access to patient medical records	3.587	0.985	0.697	0.927	No material improvement in α
Barriers	Insufficient integration with primary healthcare	3.545	1.008	0.739	0.926	No material improvement in α
Barriers	Insufficient pharmacist time	3.462	1.063	0.733	0.926	No material improvement in α
Barriers	Shortage of pharmacy assistants/technicians	3.455	1.053	0.723	0.926	No material improvement in α
Barriers	Insufficient confidence in practical skills	3.133	1.194	0.741	0.926	No material improvement in α
Barriers	Insufficient confidence in required knowledge	3.167	1.148	0.749	0.925	No material improvement in α
Barriers	Insufficient training	3.247	1.126	0.749	0.925	No material improvement in α

Supplementary Table S3. Education-Group Bivariate Tests and Effect Sizes

Education-group differences were assessed using Pearson's chi-square test when expected-cell assumptions were met and the Fisher–Freeman–Halton exact test with 100,000 Monte Carlo samples when sparse expected counts were present. Benjamini–Hochberg false-discovery-rate adjustment was applied within each questionnaire domain. Bias-corrected Cramér's V was reported as the effect-size measure, with larger values indicating stronger associations. A q-value < 0.05 was used for inference after FDR adjustment.

Domain	Item	Test	p-value	q-value	Cramér's V	Inference after FDR
Counselling	Patient counselling with every dispensing	Fisher-Freeman-Halton Monte Carlo	0.005	0.019	0.147	Significant
Counselling	Instructions on drug administration, dosage, side effects, and precautions	Pearson chi-square	0.061	0.119	0.091	Not significant
Counselling	Detection of drug–drug interactions and adverse drug reactions	Pearson chi-square	0.004	0.019	0.138	Significant
Counselling	Identification and reporting of medication errors	Pearson chi-square	0.215	0.251	0.055	Not significant
Counselling	Advice on drug therapy for chronic non-communicable diseases	Pearson chi-square	0.068	0.119	0.089	Not significant
Counselling	Counselling on non-prescription options for the common cold or influenza	Pearson chi-square	0.318	0.318	0.034	Not significant
Counselling	Medical information about diseases and medications	Pearson chi-square	0.090	0.126	0.082	Not significant
Extended services	Vaccination administration	Pearson chi-square	0.926	0.999	0.000	Not significant
Extended services	Body mass index assessment	Pearson chi-square	0.053	0.234	0.095	Not significant
Extended services	Blood cholesterol screening	Pearson chi-square	0.978	0.999	0.000	Not significant
Extended services	Blood glucose screening	Pearson chi-square	0.536	0.986	0.000	Not significant
Extended services	Blood pressure assessment	Pearson chi-square	0.999	0.999	0.000	Not significant
Extended services	Smoking-cessation counselling	Pearson chi-square	0.616	0.986	0.000	Not significant
Extended services	Emergency contraception service	Pearson chi-square	0.316	0.843	0.035	Not significant
Extended services	Advice on herbal medicines	Pearson chi-square	0.059	0.234	0.092	Not significant
Barriers	Insufficient public awareness of available services	Pearson chi-square	0.181	0.214	0.061	Not significant
Barriers	Patients or customers have insufficient time	Pearson chi-square	0.139	0.201	0.070	Not significant
Barriers	Insufficient space to protect patient privacy	Pearson chi-square	0.162	0.210	0.065	Not significant

Domain	Item	Test	p-value	q-value	Cramér’s V	Inference after FDR
Barriers	Insufficient patient demand	Pearson chi-square	0.032	0.084	0.105	Not significant
Barriers	Insufficient material resources	Pearson chi-square	0.032	0.084	0.104	Not significant
Barriers	Insufficient coordination with other healthcare professionals	Pearson chi-square	0.010	0.045	0.124	Significant
Barriers	Lack of access to patient medical records	Pearson chi-square	<0.001	<0.001	0.195	Significant
Barriers	Insufficient integration with primary healthcare	Pearson chi-square	0.087	0.142	0.083	Not significant
Barriers	Insufficient pharmacist time	Pearson chi-square	0.043	0.093	0.099	Not significant
Barriers	Shortage of pharmacy assistants/technicians	Pearson chi-square	0.053	0.098	0.095	Not significant
Barriers	Insufficient confidence in practical skills	Pearson chi-square	0.264	0.286	0.045	Not significant
Barriers	Insufficient confidence in required knowledge	Pearson chi-square	0.498	0.498	0.000	Not significant
Barriers	Insufficient training	Pearson chi-square	0.006	0.037	0.133	Significant

Supplementary Table S4. Exploratory Domain-Level Analyses

S4A. Domain-score descriptive statistics

Domain	N	Mean	SD	Median	Q1	Q3	Minimum	Maximum
Counselling	600	4.205	0.738	4.143	4.000	4.857	1.000	5.000
Extended services	600	2.118	0.869	1.875	1.500	2.500	1.000	5.000
Barriers	600	3.456	0.778	3.462	3.000	4.000	1.000	5.000

S4B. Spearman correlations among domain scores

Domain 1	Domain 2	Spearman ρ	p-value
Counselling	Extended services	0.042	0.305
Counselling	Barriers	-0.055	0.182
Extended services	Barriers	-0.312	<0.001

S4C. Exploratory robust linear model for the mean extended-service score

The model included the same demographic and practice-setting covariates used in the service-specific models, with additional adjustment for the mean counselling and perceived-barrier scores. HC3 heteroscedasticity-robust standard errors were used. For categorical predictors, adjusted mean differences are relative to the stated reference group; for the counselling and barrier scores, coefficients represent the difference associated with a one-point increase. Model $R^2 = 0.200$.

Predictor	Adjusted mean difference	95% CI lower	95% CI upper	p-value
Age 26-30 vs 20-25	0.234	0.015	0.453	0.036
Age 31-35 vs 20-25	0.154	-0.101	0.409	0.236
Age 36-40 vs 20-25	0.092	-0.186	0.369	0.518
Age >40 vs 20-25	-0.017	-0.304	0.270	0.908
Female vs Male	0.050	-0.137	0.237	0.598
Non-Saudi vs Saudi	0.077	-0.102	0.256	0.399
B.Pharm vs PharmD	-0.182	-0.390	0.026	0.086
Customers 51-100 vs 1-50	0.098	-0.061	0.257	0.227
Customers >100 vs 1-50	0.189	-0.011	0.389	0.064
Outside vs Inside Riyadh	0.358	0.208	0.507	<0.001
Chain vs Independent	0.317	0.185	0.449	<0.001
Counselling mean (per 1 point)	-0.050	-0.166	0.065	0.392
Barrier mean (per 1 point)	-0.282	-0.394	-0.170	<0.001

Supplementary Table S5. Post Hoc Sensitivity Analyses

S5A. Firth penalised logistic-regression sensitivity analysis for quasi-complete separation

Firth penalised logistic regression was fitted post hoc for the three chain-pharmacy coefficients affected by quasi-complete separation at the frequent-provision threshold, using the same covariate set as the corresponding primary adjusted models. Penalised profile-likelihood 95% confidence intervals and penalised-likelihood-ratio p-values are reported.

Frequent-provision outcome	Firth aOR: Chain vs Independent	95% penalised profile-likelihood CI	Penalised-likelihood p-value
Vaccination administration	9.17	1.23–1170.36	0.025
Blood cholesterol screening	12.79	1.75–1629.77	0.006
Blood glucose screening	16.27	2.25–2067.88	0.001

Note: For all three outcomes, no frequent-provision events were observed among independent-pharmacy respondents. Firth estimation yielded finite coefficients but extremely wide confidence intervals, indicating substantial residual imprecision due to sparse data. These estimates are presented as post hoc sensitivity analyses and do not replace the primary maximum-likelihood coefficients reported as not estimable.

S5B. Geographic sensitivity analysis of any extended-service provision by outside-Riyadh directional category

The primary geographic comparison used inside Riyadh versus outside Riyadh. As a post hoc sensitivity analysis, respondents outside Riyadh with usable directional data were separated into West, North, East, and South groups, and each group was compared with Riyadh using the same adjustment covariates as the primary any-provision models.

Extended service	Outside-Riyadh category vs Riyadh	aOR (95% CI)	p-value	q-value
Vaccination administration	West	3.50 (2.09–5.88)	<0.001	<0.001
Vaccination administration	North	1.54 (0.77–3.06)	0.223	0.266
Vaccination administration	East	2.52 (1.36–4.67)	0.003	0.006
Vaccination administration	South	2.06 (1.10–3.85)	0.023	0.037
Body mass index assessment	West	3.52 (2.04–6.10)	<0.001	<0.001
Body mass index assessment	North	3.58 (1.71–7.49)	<0.001	0.002
Body mass index assessment	East	5.76 (2.64–12.56)	<0.001	<0.001
Body mass index assessment	South	4.11 (2.02–8.37)	<0.001	<0.001
Blood cholesterol screening	West	3.60 (2.14–6.04)	<0.001	<0.001
Blood cholesterol screening	North	3.19 (1.61–6.32)	<0.001	0.002
Blood cholesterol screening	East	1.83 (0.98–3.42)	0.056	0.082
Blood cholesterol screening	South	2.81 (1.49–5.27)	0.001	0.003
Blood glucose screening	West	4.68 (2.75–7.98)	<0.001	<0.001
Blood glucose screening	North	3.99 (2.00–7.98)	<0.001	<0.001
Blood glucose screening	East	3.10 (1.65–5.79)	<0.001	0.001
Blood glucose screening	South	2.90 (1.55–5.45)	<0.001	0.002
Blood pressure assessment	West	3.39 (2.02–5.67)	<0.001	<0.001
Blood pressure assessment	North	4.76 (2.34–9.67)	<0.001	<0.001
Blood pressure assessment	East	3.73 (1.96–7.09)	<0.001	<0.001
Blood pressure assessment	South	2.74 (1.46–5.14)	0.002	0.003
Smoking-cessation counselling	West	2.03 (1.11–3.72)	0.021	0.036
Smoking-cessation counselling	North	1.62 (0.73–3.57)	0.235	0.269
Smoking-cessation counselling	East	1.95 (0.90–4.23)	0.092	0.128
Smoking-cessation counselling	South	2.21 (1.01–4.82)	0.046	0.071
Emergency contraception service	West	2.47 (1.27–4.79)	0.008	0.014
Emergency contraception service	North	1.04 (0.48–2.24)	0.922	0.922

Extended service	Outside-Riyadh category vs Riyadh	aOR (95% CI)	p-value	q-value
Emergency contraception service	East	1.63 (0.74–3.57)	0.224	0.266
Emergency contraception service	South	1.85 (0.82–4.17)	0.140	0.179
Advice on herbal medicines	West	1.55 (0.68–3.54)	0.297	0.328
Advice on herbal medicines	North	0.51 (0.22–1.18)	0.115	0.153
Advice on herbal medicines	East	1.56 (0.52–4.67)	0.424	0.453
Advice on herbal medicines	South	1.36 (0.50–3.72)	0.547	0.565

Note: Models evaluated any provision (occasionally, sometimes, mostly, or always) versus never. Riyadh was the reference location. Models were adjusted for age group, gender, nationality, educational qualification, average daily customer volume, and pharmacy type, consistent with the primary adjusted models. The analysis included 588 respondents (351 inside Riyadh and 237 outside Riyadh with usable directional information). Benjamini–Hochberg adjustment was applied across the 32 outside-Riyadh directional contrasts. The broad directional categories do not identify specific cities or locality types.
