# Supplementary material for: Self-Reported Provision of Community Pharmacy Services and Perceived Barriers in Saudi Arabia: A Cross-Sectional Survey
Source: Healthcare (Basel). 2026 Oct 13;14(20):3410. doi: 10.3390/healthcare14203410 (PMC13704151; doi:10.3390/healthcare14203410)
Supplement: Supplementary file 1 [file healthcare-14-03410-s001.zip › Supplementary File S4 - Consent and Participant-Facing Survey.pdf]

Supplementary File S4. Consent Statement and Participant-Facing Survey

A. Consent statement

Dear participant,

You are invited to participate in this survey. Your contribution is greatly valued and will help us achieve the intended purpose of this study.

Study title: Evaluation of Healthcare Services Provided by Community Pharmacists in Saudi Arabia.

Purpose of the study: To describe self-reported community pharmacy service provision in Saudi Arabia, identify perceived implementation barriers, and examine associations with pharmacist- and practice-level characteristics.

The survey will take approximately 5 minutes to complete. Participation is completely voluntary, and you may withdraw from the study at any time.

Responses are anonymous and confidential. The researchers will not be able to identify individual participants. The collected information will be used for research purposes only.

Agreement to participate:

☐ I agree to participate in this study and understand that my response will be anonymous.

☐ I do not agree to participate in this study.

If you are willing to participate in this online questionnaire-based survey, please click “Next” to begin.

B. Participant-Facing Survey Layout

Online Survey – Evaluation of Healthcare Services Provided by Community Pharmacists in Saudi Arabia

Part 1: Demographic Data	
Gender	
Male	○
Female	○
Age (years)	
20–25	○
26–30	○
31–35	○
36–40	○
More than 40 years	○
Years of community pharmacy practice experience	
1–5	○
6–10	○
11–15	○
More than 16 years	○
Average daily customer count	
1–50	○
51–100	○
More than 100 customers	○
Nationality	
Saudi	○
Non-Saudi	○
Educational background	
Bachelor of Pharmacy (B.Pharm)	○
Doctor of Pharmacy (PharmD)	○
Location of your current work	
Inside Riyadh	○
Outside Riyadh	○
If inside Riyadh, what is the location of your pharmacy?	
West	○
North	○
East	○
South	○
If outside Riyadh, what is the location of your pharmacy?	
West	○
North	○
East	○
South	○
Type of community pharmacy	
Chain	○
Independent	○

Part 2: Counselling Services					
Please indicate your level of agreement with each statement.	Strongly agree	Agree	Neither agree nor disagree	Disagree	Strongly disagree
2.1 Health and Medication Counselling Services					
1. I provide patient counselling with every dispensing.	○	○	○	○	○
2. I provide patients with instructions on drug administration, dosage, side effects, and precautions.	○	○	○	○	○
3. I detect drug–drug interactions and adverse drug reactions during counselling.	○	○	○	○	○
4. I assist in identifying and reporting medication errors to the appropriate authority.	○	○	○	○	○
5. I provide advice on drug therapy for chronic non-communicable diseases.	○	○	○	○	○
6. I provide counselling on non-prescription options for the common cold or influenza.	○	○	○	○	○
7. I provide patients with medical information about diseases and medications.	○	○	○	○	○

2.2 Other Health Services					
How frequently do you provide each of the following services?	Never	Occasionally	Sometimes	Mostly	Always
Vaccination administration	○	○	○	○	○
Body mass index assessment	○	○	○	○	○
Blood glucose screening	○	○	○	○	○
Blood pressure assessment	○	○	○	○	○
Blood cholesterol screening	○	○	○	○	○
Smoking-cessation counselling	○	○	○	○	○
Emergency contraception service	○	○	○	○	○
Advice on herbal medicines	○	○	○	○	○

Part 3: Barriers to Service Provision					
Barriers	Strongly agree	Agree	Neutral	Disagree	Strongly disagree
3.1 Patient-Related Barriers					
Insufficient public awareness that such services are available at your community pharmacy.	○	○	○	○	○
Patients or customers have insufficient time.	○	○	○	○	○
Insufficient space in the pharmacy premises to protect patient privacy.	○	○	○	○	○
Insufficient patient demand for these services.	○	○	○	○	○
3.2 Pharmacist- and Practice-Related Barriers					
Insufficient material resources, such as computers, internet access, pharmacy software, glucose-testing supplies, and needle-disposal procedures.	○	○	○	○	○
Insufficient coordination with other healthcare professionals.	○	○	○	○	○
Lack of access to patient medical records.	○	○	○	○	○
Insufficient integration of community pharmacy with the primary healthcare system.	○	○	○	○	○
Insufficient pharmacist time.	○	○	○	○	○
Shortage of pharmacy assistants or technicians.	○	○	○	○	○
Insufficient confidence in the practical skills required to provide these services.	○	○	○	○	○
Insufficient confidence in the knowledge required to interpret and discuss these services with patients.	○	○	○	○	○
Insufficient training to provide these services.	○	○	○	○	○

Thank you for taking the time to complete our survey.
